# Correlation of HIV-1 drug resistant mutations and virologic failure

**DOI:** 10.11604/pamj.2021.39.180.28818

**Published:** 2021-07-07

**Authors:** Olipher Makwaga, David Hughes Mulama, John Muoma, Matilu Mwau

**Affiliations:** 1 Kenya Medical Research Institute, Busia, Kenya,; 2 Masinde Muliro University of Science and Technology, Kakamega, Kenya

**Keywords:** HIV-1, drug resistant mutations, virologic failure

## Abstract

**Introduction:**

mutations are important by ensuring that the HIV-1 agent remains fit in the environment and evades drugs that are developed purposely to kill them. In Kenya, mutations conferring resistance to available ARVs have been reported in previous studies. However, there is a paucity of information on whether these previous studies have reported all mutations conclusively that confer resistance to available drugs leading to virologic failure. Therefore, this study was sought to identify the current HIV-1 drug-resistant mutations attributable to virologic failure among adults on various ARV regimens.

**Methods:**

the samples were collected March to June 2020. Analysis of viral loads and HIV-1 drug-resistant mutations through sequencing of the pol region of HIV-1 were done. Alignment of the cDNA sequences was done by Recall (beta version 3.05) software. HIV-1 resistant mutations were identified by Stanford University HIV drug resistance database.

**Results:**

most of the participants had viral loads of more than 1000 copies/ml during all the three visits. Out of 125 mutations identified, 83 mutations resulted in virologic failure. Out of 17 new mutations, 14 resulted in virologic failure and included NRTIs (L74I, L74V, T69D, V65R); NNRTIs (A98G, V179E, V179F, V179D, 179F); PIs (I54V3, F53L2, L89T, G48A).

**Conclusion:**

the study reveals new HIV-1 drug-resistant mutations which have never been reported in Kenya as well as old and both resulted in virologic failure. This calls for frequent monitoring and profiling of mutations that will enable decision-making in the drugs and vaccine design and development.

## Introduction

In Kenya, approximately 1.5m persons are living with HIV with annual new cases of 35000 and 6800 and deaths of 17000 and 4300 among adults and children respectively. Out of a total of 47 counties in Kenya, Busia is 5^th^ with an HIV-1 prevalence of 7.7%. ART uptake is the only option for all infected persons due to its ability to decrease HIV-1 viral tally while boosting the immune system against opportunistic infections. Nationally, about 75% and 84% of the HIV-infected adults and children are receiving ART. Consequently, a coverage uptake of ARVs in Busia is 95% and 78% among adults and children respectively [1]. Irrespective of these mileposts in the management of HIV, HIV-1 drug-resistant mutations have become a life-threatening issue because they aid the virus to evade and proliferate in the presence of drugs thus intimidating the success of management of the HIV infection.

Currently, four sets of ARVs are used to manage HIV infection in Kenya. These include NNRTIs (Nevirapine (NVP), Efavirenz (EFV)); NRTIs (Lamivudine (3TC), Abacavir (ABC), Azidovodine (AZT)), Tenofovir-disoproxil-fumarate (TDF); PIs (Lopinavir plus ritonavir (LPV/r), Atazanavir plus ritonavir (ATV/r)) and Intergrase (Dolutegravir (DTG)) [2].The development of mutations by the HIV against these single-dose treatments initially frustrated the success of management of the infection. Consequently, further development of mutations against the above drug combination regimen therapy has also become a threat to the entire management of HIV infections.

In Kenya, mutations conferring resistance to available ARVs has been reported in past studies. These mutations included M184V, K65R,D67N,K70R,K219Q,Q151M, T215F, M41L, T69N, V75M, M41L, T69N, V75M, D67G, V75M, M184I, T215N, M41LM, T215N, K219N,210W, T215Y as NRTIs; K103N/S, Y181C/Y/I/V, G190A/S, L100I,V179T, V106I/A, V108I, P225H, M230L, K238T, P225H, F227L as NNRTIs; M46I/L, D30N,M46I,V82F,L90M as PIs [3-15]. However, these studies did not link the mutations with the viral tallies and ARVs the participants were taking. Therefore, this study aimed to link the mutations with the viral tallies and ARVs the participants were taking as well as identify other new mutations encoding resistance to available classes of ARVs. This information will advance the knowledge on the evolutionary pattern of HIV-1virus in Kenya and attract a change of treatment options.

## Methods

### Study design, study site and population

The study design was cross sectional including adults of Busia attending a Comprehensive Care Clinic of Busia county referral hospital. Busia is among the 47 counties of Kenya. Busia county is number five in the country with the highest prevalence of HIV. Moreover, its prevalence is higher than nationwide [11]. It is situated alongside the Kenya-Uganda boundary. Busia county referral hospital where the study was conducted, is a reference level four hospital in the region with a high capacity hospital patient attendance. The hospital is similarly among the health facilities which benefits from the Academic Model Providing Access to Healthcare (AMPATH) Integrating Nutrition Support initiative where recently HIV infected individuals are aggressively registered for ARVs and old ones are reserved on ARVs by providing food to HIV infected persons and their dependents [14]. AMPATH is an academic medical partnership between North American academic health centers led by the Indiana University School of Medicine in Indianapolis, Indiana and the Moi University School of Medicine, centered in Eldoret, Kenya. The participants who were included in the study were HIV-1 infected and taking ARVs treatment, were aged ≥ 18 years and consented to participate.

**Sample size determination:** a formula by [16] was used to compute the minimum number of samples:

$$n=\frac{4{\left(\text{Zcrit}\right)}^{2}\text{P(1-p)}}{d^{2}}$$

n = minimum sample size; Zcrit is the standard normal deviation = 1.96 (standard errors from the mean) at D = 0.05% level of significance, p = prevalence of infection. The prevalence of 1.1% of HIV Type 1 drug-resistance among adults in a rural HIV clinic in Kenya [5] being too low for this study, we averaged these by an overall prevalence of 4.7% of drug-resistant HIV-1 transmission in Africa [16] to compute our sample size and this was 2.9%. p= 0.029 HIV-1 drug resistance prevalence, therefore, n= 43 blood samples which were rounded to 50.

### Sampling procedure

All adult participants attending a comprehensive HIV care Clinic of Busia county-referral hospital from the months of March to June 2020, already taking ARVs and consented to participate in the study were recruited systematically. Demographics (age and sex) and duration on ARVs were collected through interview using a simple questionnaire by a qualified health officer. The questionnaire was written in both English and Swahili which are both the national languages for Kenya. The clinicians requested the participants to decide on one language they were very much conversant with so that the correct information could be collected. About 5 ml of blood samples were collected using sterile needles and syringes in EDTA tubes by qualified medical laboratory Technologists. Triple packaging of the samples was done and transported to KEMRI laboratory following the national sample consignment program for clinical specimens. Samples were removed from the cold environment and allowed to come to room temperature for at least one hour before processing.

### Viral load and HIV-1 drug-resistance testing

Samples for analyzing viral load were collected in plasma preparation tubes (Becton-Dickinson, San-Jose, CA-USA). Plasma were mixed into cryo-tubes. Extraction and quantification of RNA was carried out in Abbott real time machine (Abbott Molecular Inc. USA) and Cobas-Ampliprep/ Cobas-Taqman HIV-1 test v.2.0 (Roche Diagnostic, USA) according to manufacturer´s procedures [17]. Transcription of RNA into cDNA was carried out using an in-house reverse-transcriptase polymerase chain reaction (RT-PCR) protocol by Thermo Fisher Scientific´s Genotyping kit. The extracted-RNA was denatured at 65°C for 10 minutes. The RT -PCR was carried out following these cycling environments: 1 cycle of reverse-transcription at 500C for 45 minutes, 1 cycle of enzyme inactivation at 940C for 2 minutes, 40 cycles of denaturation at 940C for 15 seconds, 40 cycles of annealing at 500C for 20 seconds, 40 cycles of extension at 720C for 2 minutes and one cycle of final extension at 720C for 10 minutes. The nested PCR was done under the following cycling conditions: one cycle of initial denaturation at 940C for 4 minutes, 40 cycles of denaturation at 940C for 15 seconds, 40 cycles of annealing at 550C for 20 seconds, 40 cycles of extension at 720C for 2 minutes and one cycle of final extension at 720C for 10 minutes. Agarose gel (1%) was used to confirm the PCR amplified results. Gel electrophoresis was done for confirmation of the amplified DNA by the gel visualization on the imaging system and photographed. Purification of the PCR products was performed using Thermo Fisher Scientific´s Clean Sweep Purification Reagent under the following conditions: Digest at 370C for 15 minutes and heat deactivation at 800C for 15 minutes. Cycle sequencing was done using 6 sequencing mixes (F1, F2, F3, R1, R2, and R3) and PGEM sequencing control. The cycle sequencing conditions was set at 25 cycles of denaturation, annealing and extension at 96°C for 10 seconds, 50°C for 5 seconds, and 60°C for 4 minutes respectively. Purification of cycle sequencing products was done using Thermo Fisher Scientific´s Big Dye X-Terminator Purification Kit. Direct sequencing of the pol gene encoding protease (codons 6-99) and reverse transcriptase (codons 1-239) was done on the 3730XL DNA Analyzer (Applied Biosytems) using Sanger sequencer. Base calling was facilitated by Seq Scanner v.6 (Applied Bio systems Inc. USA). This was by evaluating the main technical parameters (i.e., raw data, electropherograms and the quality value of sequenced bases). RECall (beta v3.05) software (http: //pssm.cfenet.ubc.ca/) was used for aligning and generating the consensus sequences and Drug-resistance was determined using the International Aids Society (IAS) algorithm and the Stanford University HIV database.

### Ethical approval and considerations

All ethical processes were considered. Scientific and Ethical clearance for this study was sought from the national ethical review committee at Kenya Medical Research Institute (KEMRI) which issued a study number KEMRI/SERU/CIPDCR/008/3333. This study involved the analysis of clinical specimens. Blood samples were collected and coded without using any participant´s identifier. Other relevant information was collected orally on a simple questionnaire by qualified health officers in Busia referral hospital. These questionnaires were kept under lockable cabinets and accessed only by Investigators. The samples were taken to KEMRI laboratory for PCR and sequencing using the recommended national courier services in the country. The results were interpreted by investigators in a language that the clinicians understood. All the soft data and write ups of this study had password protected so as to be accessed only by Investigators. Thereafter, both original and interpreted results were sent back to the health facility using courier services so that they are released to clinicians and participants. The results of this study enabled the participants to have information whether the drugs they are taking are working well or not and why they may be changed to other ARVs. Clinicians used these results to manage participants appropriately.

### Data management and statistical analysis

Collected data was entered, cleaned and stored in excel in columns of variables in a password protected desktop and only accessible to Investigators. Hard disk and external drive was used to back up the data. Analysis of data was conducted by employing SPSS v. 20 (Armonk NY: IBM, Corp). The key variables were HIV-1 drug resistant mutations and viral tallies. Descriptive measurements were carried out to analyze socio demographic characteristics and the frequency of various HIV-1 drug-resistant mutations. Viral tallies generated by the Cobas-Ampliprep/ Cobas-Taqman were converted into log10 using a scientific calculator. Pearson´s correlation was used to link the viral tallies and various mutations, whereby a negative and a positive figure marked the relationship. Paired sample t test was used to establish a p value between mutations and the viral tallies of which a p value of less than 0.05 was termed as significant. Systematic reviews of 200 related papers to this study in Kenya were identified through MEDLINE and EMBASE of which 23 were eligible for inclusion in the review and five from other sources.

## Results

About 50 (female, 31 and male, 19) participants were included in the current study. Their age range was from 18 to 66 years old (median age 35; interquartile ranges 28 years). Many participants were on AZT+3TC+NVP and TDF+3TC+EFV ARVs regimen combination (Table 1). Table 2 shows correlations between specific mutations, ARV regimen the participants were taking, and viral loads of the three consecutive visits of 34 participants whose samples had mutations. Each specific mutation conferring resistance to a certain drug of a given participant is highlighted with the same color. Some mutations existed which did not confer resistance to the drugs the patients were taking. This does not mean that the mutations are not important; they are because they confer resistance to other drugs as per the Stanford University HIV drug resistance database. Higher viral loads in all participants persisted. Most of the old mutations identified in the previous studies encoded resistance to prescribed drugs causing virologic failure. Most of the new mutations also encoded for resistance to prescribed drugs and these included L74I/V, T69D, V65R as NRTIs; A98G, V179E/F/D/F as NNRTs and I54V, F53L, L89T, G48A, K20T as PIs. Pearson´s correlation; r = 0.311 and a p value of 0.028 between viral tallies and all the mutations showed a strong positive relationship between these two variables. Thus participants are likely to have mutations with the increasing viral loads in the presence of ARVs uptakes.

**Table 1:** gender and ARVs regimen combination enrolled participants were taking, n = 50

Gender	ABC. 3TC. EFV.	ABC. 3TC. LPV/r.	AF5X-OTHER	AZT. 3TC. ATV/r	AZT. 3TC. NVP	TDF. 3TC. ATV/r	TDF. 3TC. DTG	TDF. 3TC. EFV	TDF. 3TC. LPV/r	TDF. 3TC. NVP	Total
Female	1	1	0	6	8	2	1	10	1	1	31
Male	1	0	1	2	3	2	2	7	0	1	19
Total	2	1	1	8	11	4	3	17	1	2	50

n= number of samples/participants sampled; log10=logarithm of 10; ARV=Antiretroviral; AZT=Azidovudine; 3TC= Lamivudine; EFV= Efevirenz; LPV/r= Lopinavir/ritonavir; ATV/r= Atazanavir/ ritonavir; NVP= Nevirapine; TDF= Tenofovir disoproxil fumarate; DTG= Dolutegravir.

**Table 2:** correlation of mutations identified and viral loads of participants on various ARVs, n = 34

No	Viral Load1 log10 copies/ml	Viral load2 log10 copies/ml	Viral load3 log10 copies/ml	ARV regimen	NRTI mutation	NNRTI mutation	PI mutation
1	4.9	4.85	4.96	AZT.3TC.ATV/r	M184V,D67N, K219E,215F	G190A	I54V,G48V,V82A
2	4.74	4.7	4.73	TDF.3TC.DTG	M184I,K70E	Y181C,K101E	None
3	5.48	5.45	5.42	AZT.3TC.ATV/r	M184V,L74I	K103N,A98G,P225H	None
4	3.85	3.7	3.7	3TC.AZT. TV/r	M184V, V75I	H221Y, Y181C	None
5	3.7	3.7	3.76	3TC.AZT. TV/r	T215F, M184V	E138A, K103N	F53L, M46I, L89T
6	5.4	5.4	5.42	3TC.AZT. EFV	M184V,L74V, Y115F	V179E,K103N, Y181C	None
7	4.35	4	4.1	3TC.TDF.ATV/r	T215Y,M184V, M41L	-	I54V,G48A, Q58E, V82S
8	4.95	5	5	TDF.3TC.EFV	-	-	I54V
9	2.82	5.17	4.42	TDF.3TC.EFV	-	K103N	-
10	4	4.01	4.03	AZT.3TC.NVP	M184V	K103N	-
11	2.9	4.43	3.43	AZT.3TC.NVP	-	K103S	-
12	4.94	4.35	4.6	AZT.3TC.ATV/r	,M184V,D67NK70E	E138A,K101E, G190S	K20T
13	3.85	3.85	3.89	AZT.3TC.EFV	-	K103N	-
14	5.11	5.16	5.17	TDF.3TC+EFV	-	-	F53L
15	3.46	4.35	4.4	TDF+3TC.EFV	K70T,D67N, T69D	-	-
16	2.9	4.87	4.73	AZT.3TC.ATV/r	Y115F, K65R	Y181C ,H221Y,K103N,V179F	-
17	4.2	4.2	4.27	TDF.3TC.ATV/r	K70E	P225H,K103N	-
18	4.73	4.75	4.76	TDF.3TC.ATV/r	M184V	E138A, G190A,K103N	-
19	3.5	1.7	4.66	AZT.3TC.NVP	M184V	H221Y ,Y181C	-
20	4	4.09	3.7	TDF.3TC.EFV	M184V, K65R	M230L,K103N	-
21	1.7	3.36	4.32	AZT.3TC.EFV	-	G190A,K101E,V179D	-
22	4.18	4.26	4.12	TDF.3TC.EFV	Y115F, K65R	Y181C,H221Y, K103N,V179F	-
23	4.11	4.3	5.62	AZT.3TC.ATV/r	M84V	Y181I	-
24	5.11	5.15	5.17	TDF.3TC.EFV	-	K101E	-
25	4.29	3.85	4.03	AZT.3TC.EFV	M184V,L74V,Y115F	G190A,K103N	-
26	4.48	3.4	4.45	AZT.3TC.EFV	M184V	V108I,K103N, M230L	-
27	1.81	4.27	4.82	AZT.3TC.EFV	Y115F,K65R, M184V	Y188L,V106I	-
28	5.3	4.66	5.29	AZT.3TC.NVP	-	G190A	-
29	3.72	4.27	4.19	AZT.3TC.LPV/r	M184V	G190A,K101H, P225H	V82A,I54V, L10F
30	3.3	4.71	4.76	AZT.3TC.LPV/r	-	K103N	-
31	2.37	3.78	3.84	AZT.3TC.NVP	-	H221Y,Y181C	-
32	4.09	2.1	5.09	AZT.3TC.NVP	M184V	-	-
33	2.95	4.83	4.95	TDF.3TC.ATV/r	V65R, A62V	Y181C,K101E,A98G, G190A	-
34	3.73	4.48	4.67	AZT.3TC.NVP	-	K103N	-

n= number of samples/participants with mutations; NNRTIs= non and nucleotide reverse transcriptase inhibitors; PIs= protease inhibitors; log10=logarithm of 10; ARV=Antiretroviral; AZT=Azidovudine; 3TC= Lamivudine; EFV= Efevirenz; LPV/r= Lopinavir/ritonavir; ATV/r= Atazanavir/ ritonavir; NVP= Nevirapine; TDF= Tenofovir disoproxil fumarate; DTG= Dolutegravir. Pearson’s correlation between viral load and mutations was r = 0.311 with a p value of 0.028among participants on various ARVs. This signifies a strong relationship between the two variables.

## Discussion

The importance of ARV treatment in HIV infected persons is massive as it prolongs their survival, advances their value of life and minimizes the morbidity and mortality rates [18]. ARV treatment is the standard care for all HIV infected persons in Kenya [19]. However, there is a contest due to HIV drug-resistant mutations which weakens effectiveness of the drug. Subsequently, HIV-1 drug-resistance testing is significant in resolution making concerning the management of HIV infected persons [20]. However, HIV-1 drug-resistance tests are not routinely available in the country and are only carried out in limited research laboratories in Kenya. The study was planned to identify both old and new mutations that confer resistance to the drugs participants were taking in relation to virologic failure. Additional HIV infection was regularly detected in women more than men, endorsing past studies [10, 13] that the prevalence of HIV among women is more than that of men in the country and that men are poor health pursuers compared to women. Further analyses demonstrated that the proportion of women to men in this study was similar, giving a podium of gender assessment as it is reported with National AIDS and STI Control Program (NASCOP) that fairly more women are infected with HIV than men [2].

In broad, the study determined the current HIV-1 drug-resistant mutations attributable to virologic failure among adults on various ARV regimens. The study noted the current drug regimen combinations which the participants were taking. Additionally, the viral loads for the three consecutive visits were determined. Further, the specific mutations encoding for resistance to various classes/types of ARVs were analyzed.

In the current study, participants were taking 10 diverse regimens of ARVs that are regularly accessible. Additional analysis showed that many participants were taking TDF+3TC+EFV and AZT+3TC+NVP as first line HIV-1 treatment because the former regimen is standby first line ART regimen and the latter is ideal first line ART regimen in pregnancy. These outcomes concur with the fact that many women were put on these two drugs than other regimens of ARVs. This study did not investigate pregnancy history. AZT+3TC+ATV/r was being taken by many participants compared to other second line drugs, perhaps due to the guidelines that after failure on the above ideal and substitute of first line drugs, the patient be switched to AZT+3TC+ATV/r as a second-line drug [21], evidenced by our study that more participants were taking the two first line drugs compared to the rest. Most of the participants had persisting higher viral tally during all the visits. This was a sign of virologic failure which was justified by the presence of multiple mutations in the samples of the participants.

Previous related studies [3-15] identified some of the mutations that were identified in this study, however, our study reports other several new mutations of clinical importance which were not reported in the previous studies. The existence of these new mutations that encode resistance to the present drugs the participants are currently undertaking. Furthermore, the strong positive relationship between the viral loads and mutations displayed in this study intimidates the entire management of HIV infection. Therefore, the current data increases evidence to the earlier literature on HIV-1 drug-resistance. It also informs in the verdict making regarding the treatment selections midst diseased persons. Information in this study pleas for strengthening of the health systems in the Country.

**Limitation of the study:** our study concentrated on the determining of HIV-1 drug-resistant mutations by sequencing the pol gene. This would underrate the general mutations. This is because some drugs mark the gag as well as envelope genes of the HIV. Therefore, we recommend in such situations, all the three genes to be sequenced.

## Conclusion

The study reveals new HIV-1 drug-resistant mutations which have never been reported in Kenya as well as old and both result to virologic failure. This calls for frequent monitoring and profiling of mutations that will enable decision-making in the drug design and development.

### What is known about this topic

- HIV drug-resistance is a threat to the general population;
- Presently, HIV infected individuals entirely are started on treatment irrespective of their CD4 counts;
- ART uptake is increasing in all the Kenyan counties.

### What this study adds

- Data collected in this study increments information to the previously existing literature on HIV-1 drug-resistance;
- Evidence in this study will enlighten in the decision making regarding treatment choices amongst infected individuals;
- Materials in this study calls for health systems strengthening in the county and in the entire Country.
